# Osteopathic manipulative treatment for low back pain: a systematic review and meta-analysis of randomized controlled trials

**DOI:** 10.1186/1471-2474-6-43

**Published:** 2005-08-04

**Authors:** John C Licciardone, Angela K Brimhall, Linda N King

**Affiliations:** 1 Osteopathic Research Center, University of North Texas Health Science Center, Fort Worth, TX 76107, USA; 2 Department of Family Medicine, University of North Texas Health Science Center, Fort Worth, TX 76107, USA; 3 Gibson D. Lewis Health Science Library, University of North Texas Health Science Center, Fort Worth, TX 76107, USA

## Abstract

**Background:**

Osteopathic manipulative treatment (OMT) is a distinctive modality commonly used by osteopathic physicians to complement their conventional treatment of musculoskeletal disorders. Previous reviews and meta-analyses of spinal manipulation for low back pain have not specifically addressed OMT and generally have focused on spinal manipulation as an alternative to conventional treatment. The purpose of this study was to assess the efficacy of OMT as a complementary treatment for low back pain.

**Methods:**

Computerized bibliographic searches of MEDLINE, EMBASE, MANTIS, OSTMED, and the Cochrane Central Register of Controlled Trials were supplemented with additional database and manual searches of the literature.

Six trials, involving eight OMT vs control treatment comparisons, were included because they were randomized controlled trials of OMT that involved blinded assessment of low back pain in ambulatory settings. Data on trial methodology, OMT and control treatments, and low back pain outcomes were abstracted by two independent reviewers. Effect sizes were computed using Cohen's *d *statistic and meta-analysis results were weighted by the inverse variance of individual comparisons. In addition to the overall meta-analysis, stratified meta-analyses were performed according to control treatment, country where the trial was conducted, and duration of follow-up. Sensitivity analyses were performed for both the overall and stratified meta-analyses.

**Results:**

Overall, OMT significantly reduced low back pain (effect size, -0.30; 95% confidence interval, -0.47 – -0.13; P = .001). Stratified analyses demonstrated significant pain reductions in trials of OMT vs active treatment or placebo control and OMT vs no treatment control. There were significant pain reductions with OMT regardless of whether trials were performed in the United Kingdom or the United States. Significant pain reductions were also observed during short-, intermediate-, and long-term follow-up.

**Conclusion:**

OMT significantly reduces low back pain. The level of pain reduction is greater than expected from placebo effects alone and persists for at least three months. Additional research is warranted to elucidate mechanistically how OMT exerts its effects, to determine if OMT benefits are long lasting, and to assess the cost-effectiveness of OMT as a complementary treatment for low back pain.

## Background

Historically, low back pain has been the most common reason for visits to osteopathic physicians [1]. More recent data from the Osteopathic Survey of Health Care in America has confirmed that a majority of patients visiting osteopathic physicians continue to seek treatment for musculoskeletal conditions [2,3]. A distinctive element of low back care provided by osteopathic physicians is osteopathic manipulative treatment (OMT). A comprehensive evaluation of spinal manipulation for low back pain undertaken by the Agency for Health Care Policy and Research in the United States concluded that spinal manipulation can be helpful for patients with acute low back problems without radiculopathy when used within the first month of symptoms [4]. Nevertheless, because most studies of spinal manipulation involve chiropractic or physical therapy [5], it is unclear if such studies adequately reflect the efficacy of OMT for low back pain.

Although the professional associations that represent osteopaths, chiropractors, and physiotherapists in the United Kingdom developed a spinal manipulation package consisting of three common manual elements for use in the UK Back pain Exercise and Manipulation (UK BEAM) trial [6], there are no between-profession comparisons of clinical outcomes [7,8]. It is well known that OMT comprises a diversity of techniques [9] that are not adequately represented by the UK BEAM trial package. Professional differences in spinal manipulation are more pronounced in research studies, where chiropractors have focused almost exclusively on high-velocity-low-amplitude techniques [10]. For example, a major trial of chiropractic manipulation as adjunctive treatment for childhood asthma used a high-velocity-low-amplitude thrust as the active treatment [11]. The simulated treatment provided in the sham manipulation arm of this chiropractic trial, which ostensibly was thought to have no therapeutic effect, had a marked similarity to OMT [10,12]. Further, because differences in professional background and training lend themselves to diverse manipulation approaches, clinicians have been warned about generalizing the findings of systematic reviews to practice [13].

In addition to professional differences in the manual techniques themselves, osteopathic physicians in the United States, unlike allopathic physicians, chiropractors, or physical therapists, can treat low back pain simultaneously using both conventional primary care approaches and complementary spinal manipulation. This represents a unique philosophical approach in the treatment of low back pain. Consequently, there is a need for empirical data that specifically address the efficacy of OMT for such conditions as low back pain [14]. The present study was undertaken to address this need by conducting a systematic review of the literature on OMT and performing a meta-analysis of all randomized controlled trials for low back pain performed in ambulatory settings.

## Methods

### Search

A search of the English language literature was performed through August 2003 to identify reports of randomized controlled trials of OMT. We searched MEDLINE, OLDMEDLINE, EMBASE, MANTIS, OSTMED, Alt Health Watch, SciSearch, ClinicalTrials.gov, CRISP, and the Cochrane Central Register of Controlled Trials. A detailed description of the search strategy is provided in the Appendix [see Additional file 1]. Additionally, reports were sought from relevant reviews or meta-analyses of spinal manipulation [9,15-32] and manual searches of reference citations in the reviewed literature sources.

### Selection

The search bibliographies and relevant reports were reviewed by the authors to identify randomized controlled trials involving OMT in human subjects. To assess the efficacy of OMT in primary care, eligibility was limited to randomized controlled trials of OMT performed by osteopaths, osteopathic physicians, or osteopathic trainees that included blinded assessment of low back pain in ambulatory settings. Trials that involved manipulation under anesthesia, industrial settings, or hospitalized patients were not included. Because there is potential confusion regarding the type of manipulation performed in some trials [33], the reported methods in each trial were carefully reviewed to assess eligibility for the meta-analysis. Overall, seven studies known or purported to involve OMT for low back pain were reviewed and excluded for not meeting all eligibility criteria [34-40]. A subsequent source [41] indicated that an osteopathic manipulation technique was used in the Irvine study [42]. Although several of the six included OMT trials were identified in multiple bibliographic databases, five [42-46] were indexed in MEDLINE. The Cleary [47] trial was identified exclusively through the Cochrane Central Register of Controlled Trials.

### Data extraction

Each eligible trial was independently reviewed by two of us to abstract data on methodological characteristics, OMT and control treatments, and low back pain outcomes. Conflicting data were resolved by consensus.

### Trial characteristics

As shown in Table 1, the six OMT trials were conducted between 1973 and 2001 in the United Kingdom or the United States [42-47]. Two of the six trials each included two control treatments [43,46], thus providing eight OMT vs control treatment comparisons. The trials generally were comparable in their methodology, with the possible exception of the Cleary [47] trial. Twenty contrasts were reported in the six trials (a contrast refers to a within-trial comparison between OMT and a control treatment with respect to a low back pain outcome at a given point in time). Following randomization, nine contrasts were reported within one month (short-term outcomes), another seven contrasts were reported within three months (intermediate-term outcomes), and the remaining four contrasts were reported within 12 months (long-term outcomes).

**Table 1:** Summary of trials.

	**Hoehler 1981 [42]**	**Gibson 1985 [43]**	**Cleary 1994 [47]**
			
**Years conducted**	1973–1979	...	1991–1992
**Country**	United States	United Kingdom	United Kingdom
**Setting**	University clinic	Hospital outpatient clinic	Ambulatory clinic
**No. of subjects randomized**	95	109	30*
**Comparison**	OMT vs soft tissue massage and sham manipulation	OMT vs short-wave diathermy OMT vs detuned short-wave diathermy	OMT vs sham manipulation
**Subject** **characteristics**			
Age, y			
Mean ± SD	OMT, 30.1 ± 8.4 Controls, 32.1 ± 9.8	OMT, 34 ± 14 Short-wave diarthermy controls, 35 ± 16 Detuned short-wave diathermy controls, 40 ± 16	Overall age range, 50–60
Sex			
% male	OMT, 59 Controls, 59	OMT, 49 Detuned short-wave diathermy controls, 68 Short-wave diarthermy controls, 53	OMT, 0 Controls, 0
Type of low back pain	Referred patients with acute or chronic low back pain	Referred patients with low back pain of greater than 2 months' and less than 12 months' duration	Recruited subjects with chronic low back pain in conjunction with menopausal symptoms
**OMT protocol**			
Technique	High-velocity, low-amplitude thrust only	Variety of techniques	Low-force techniques
No. of treatments			
Mean ± SD	OMT, 4.8 ± 2.7 Controls, 3.9 ± 2.5	4, per protocol	10, per protocol
**Outcomes assessment**	Blinded	Blinded	Assessment independent of treatment, blinding not specified
**No. of pain contrasts**	3	6 (3 for each of the two OMT vs control treatment comparisons)	1
**Type of pain outcome**	Dichotomous pain outcomes	Dichotomous pain outcomes	Dichotomous pain outcome
**Timing of pain contrasts**			
Short-term	First treatment and mean, 20–30 days following randomization	2 and 4 weeks	...
Intermediate-term	Mean, 41–51 days following randomization	...	...
Long-term	...	12 weeks	15 weeks
	**Andersson 1999 [44]**	**Burton 2000 [45]**	**Licciardone 2003 [46]**
			
**Years conducted**	1992–1994	...	2000–2001
**Country**	United States	United Kingdom	United States
**Setting**	Health maintenance organization	Hospital orthopedic department	University clinic
**No. of subjects randomized**	178	40	91
**Comparison**	Usual care and OMT vs usual care only	OMT vs chemonucleolysis	Usual care and OMT vs usual care and sham manipulation Usual care and OMT vs usual care only
**Subject characteristics**			
Age, y			
Mean ± SD	OMT, 28.5 ± 10.6 Controls, 37.0 ± 11.0	Overall, 41.9 ± 10.6	Usual care and OMT, 49 ± 12 Usual care and sham manipulation controls, 52 ± 12 Usual care only controls, 49 ± 12
Sex			
% male	OMT, 41 Controls, 44	Overall, 48	Usual care and OMT, 31 Usual care and sham manipulation controls, 43 Usual care only controls, 35
Type of low back pain	Patients with low back pain of 3 or more weeks' and less than 6 months' duration	Recruited patients with low back pain and sciatica; mean duration, 30 and 32 weeks in OMT and chemonucleolysis groups, respectively	Recruited subjects with low back pain of at least 3 months' duration
**OMT protocol**			
Technique	Variety of techniques, individualized to patient	Variety of techniques, individualized to patient	Variety of techniques, individualized to subject
No. of treatments			
Mean ± SD	8, per protocol	Mean for OMT, 11; range 6–18	7, per protocol
**Outcomes assessment**	Blinded	Blinded	Blinded
**No. of pain contrasts**	1	3	6 (3 for each of the two OMT vs control treatment comparisons)
**Type of pain outcome**	Pain scale	Pain scales	Pain scales
**Timing of pain contrasts**			
Short-term	...	2 weeks	1 month
Intermediate-term	12 weeks	6 weeks	3 months
Long-term	...	12 months	6 months

OMT denotes osteopathic manipulative treatment.

*A total of 30 subjects with menopausal symptoms were randomized; however, only 12 subjects had low back pain.

The methodological quality of four of the OMT trials [42-45] was independently confirmed in a recent systematic review that included a best evidence synthesis incorporating eight explicit quality criteria, including similarity of baseline characteristics of subjects or reporting of adjusted outcomes; concealment of treatment allocation; blinding of subjects; blinding of providers or other control for attention bias; blinded or unbiased outcomes assessment; subject dropouts reported and accounted for in the analysis; missing data reported and accounted for in the analysis; and intention-to-treat analysis or absence of differential co-interventions between groups in studies with full compliance [13]. The Cleary [47] trial was not eligible for this review because it did not include a sufficiently large number of subjects. Although the Licciardone [46] trial was not eligible for the review because it was published after the closing date of December 2002, it has been characterized as an innovative and important trial with many rigorous design features [48], and more recently has been identified as an evidence-based supplement relative to the previous review from the Cochrane Library [49].

### Quantitative data synthesis

We used the effect size, computed as Cohen's *d *statistic, to report all trial results [50]. A negative effect size represented a greater decrease in pain among OMT subjects relative to control treatment subjects. Dichotomous pain measures were transformed to effect sizes by first computing the relevant P-value and then determining the effect size and 95% confidence interval (CI) that would obtain under the assumption of a two-tailed *t*-test for measuring the standardized mean difference between OMT and control treatments in the relevant number of subjects [50]. The meta-analysis results were weighted by the inverse variance for each OMT vs control treatment comparison. The *Q *statistic was used to test the homogeneity of trials included in each analysis [51].

The overall meta-analysis included the eight OMT vs control treatment comparisons. Four of the six trials, involving six of the eight OMT vs control treatment comparisons, each reported three contrasts [42,43,45,46] (Table 1). The median contrast, as identified by the intermediate effect size among the three reported pain outcomes for a given OMT vs control treatment comparison, was used to represent the pain outcome for each of these six comparisons. These median contrasts were then combined with the lone contrasts reported in each of the two remaining OMT vs control treatment comparisons [44,47]. Based on the similarity among trials (Table 1), a fixed-effects model initially was used to perform meta-analysis and the results were then compared with those of a random-effects model.

A series of sensitivity analyses were then performed. First, to address the possibility of bias by using the median contrasts method, analyses were repeated using the best-case and worst-case scenarios for the six relevant OMT vs control treatment comparisons [42,43,45,46]. Second, to address the possibility of bias by including comparisons involving the same OMT group vs two different control treatment groups in two trials [43,46], analyses were repeated using only one OMT vs control treatment comparison for each of these trials. Each of the four possible combinations of contrasts was analyzed. Third, the analysis was repeated after excluding the Cleary [47] trial. Finally, an analysis was performed using all 20 low back pain contrasts. Similar analyses were performed after stratifying trials according to control treatment, country where the trial was performed, and duration of follow-up.

As summarized in Table 2, there were 43 analyses performed, including the overall meta-analysis, seven stratified meta-analyses, and 35 sensitivity analyses. Meta-analysis was performed only when there were at least three contrasts available for data synthesis. Database management and analyses were performed using the Comprehensive Meta-Analysis software package (Version 1.0.23, Biostat, Inc, Englewood, NJ 07631, USA).

**Table 2:** Summary of analyses.*

**Meta-Analyses**	**Sensitivity Analyses**
	
***Overall Median Contrasts***	Best-case and worst-case scenarios 4 possible combinations of contrasts including one control treatment per trial Cleary [47] trial excluded All 20 contrasts
***Stratified Median Contrasts***	
***A. Control Treatment***	
1. Active treatment or placebo control	Best-case and worst-case scenarios 2 possible combinations of contrasts including one control treatment per trial Cleary [47] trial excluded All 16 contrasts
2. No treatment	
***B. Country Where Trial was Performed***	
3. United Kingdom	Best-case and worst-case scenarios 2 possible combinations of contrasts including one control treatment per trial Cleary [47] trial excluded All 10 contrasts
4. United States	Best-case and worst-case scenarios 2 possible combinations of contrasts including one control treatment per trial All 10 contrasts
***C. Duration of Follow-Up***	
5. Short-term	Best-case and worst-case scenarios All 9 contrasts
6. Intermediate-term	4 possible combinations of contrasts including one control treatment per trial
7. Long-term	2 possible combinations of contrasts including one control treatment per trial Cleary [47] trial excluded

*There were insufficient contrasts to perform sensitivity analyses for the no treatment stratified analysis. For the short-term stratified analysis, the median contrast was defined to be that corresponding to the eighth combination when effect sizes for the 16 possible contrast combinations were rank ordered from least to greatest. For the intermediate-and long-term stratified analyses, the median contrasts defaulted to the all-contrasts analyses because there were no repeated measures within these time intervals in any trial. All possible contrast combinations were included in the sensitivity analyses for intermediate-and long-term follow-up because of the limited numbers of combinations for these analyses.

## Results

### Overall analyses

**Figure 1:**
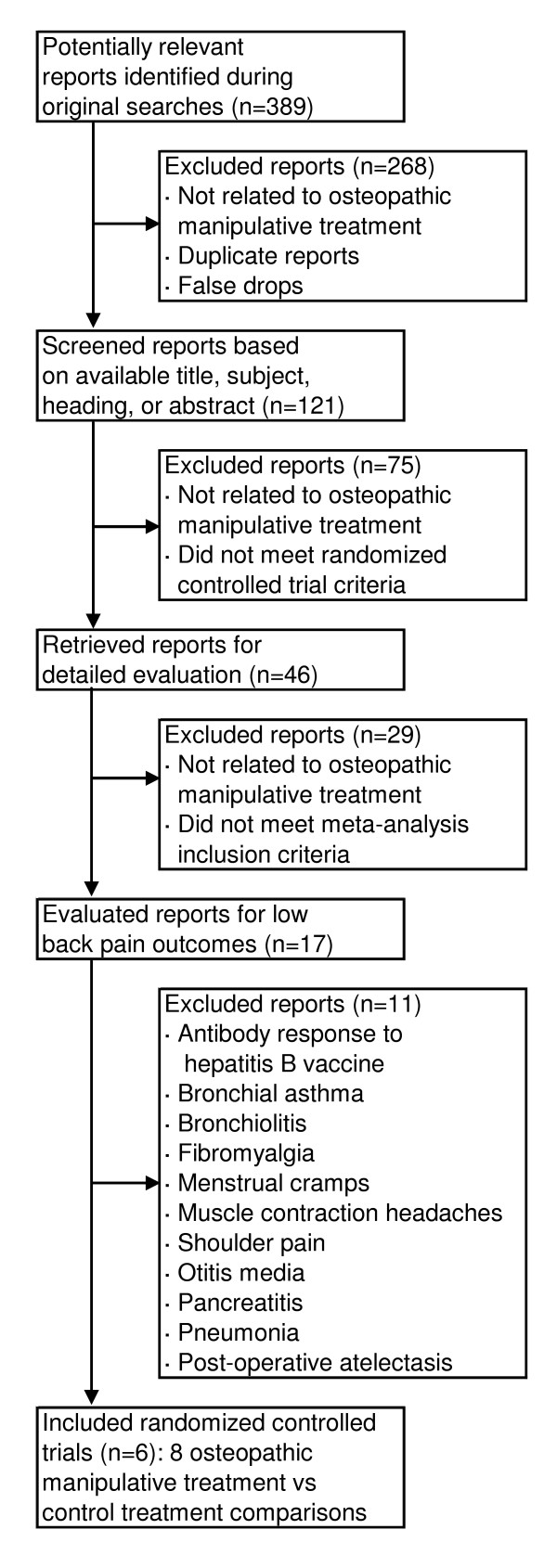
Flowchart of trials.

**Figure 2:**
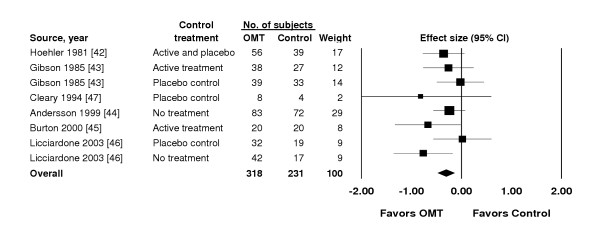
Effect size for low back pain. CI denotes confidence interval; OMT, osteopathic manipulative treatment. Overall effect size, -0.30; 95% CI, -0.47 – -0.13; P = .001.

The search for reports is summarized in Figure 1. A total of 525 subjects with low back pain were randomized in the eligible trials. The overall results are presented in Figure 2. There was a highly significant reduction in pain associated with OMT (effect size, -0.30; 95% CI, -0.47 – -0.13; P = .001). The *Q *statistic was non-significant, thus supporting the assumption of homogeneity among trials. The primary sensitivity analyses are presented in Table 3. Using a random-effects model, the results were virtually identical to those observed with a fixed-effects model. There were 729 (3^6 ^× 1^2^) possible combinations of contrasts for analysis based on three contrasts for each of six OMT vs control treatment comparisons [42,43,45,46] and one contrast for each of the two remaining OMT vs control treatment comparisons [44,47]. The efficacy of OMT for low back pain was supported in both the best-case (effect size, -0.37; 95% CI, -0.55 – -0.20; P < .001) and worst-case (effect size, -0.18; 95% CI, -0.35 – 0.00; P = .046) scenarios. Similarly, when each trial was limited to one OMT vs control treatment comparison, OMT was found to be efficacious in each of the four analyses. OMT also demonstrated significantly greater low back pain reduction than control treatment in analyses with the Cleary [47] trial excluded and with all 20 contrasts included.

**Table 3:** Overall results.

		**No. of Subjects**				
					
**Model**	**No. of** **Contrasts**	**OMT**	**Control**	**Effect** **Size**	**95% CI**	**P**
Median contrasts						
Fixed-effects model*	8	318	231	-0.30	-0.47 – -0.13	.001
Random-effects model	8	318	231	-0.31	-0.49 – -0.13	.001
Best-case scenario	8	293	220	-0.37	-0.55 – -0.20	<.001
Worst-case scenario	8	298	221	-0.18	-0.35 – 0.00	.046
Median contrasts, one OMT vs control treatment comparison per trial						
Gibson [43] active treatment control and Licciardone [46] placebo control	6	237	181	-0.30	-0.49 – -0.10	.003
Gibson [43] active treatment control and Licciardone [46] no treatment control	6	247	179	-0.39	-0.59 – -0.20	<.001
Gibson [43] placebo control and Licciardone [46] placebo control	6	238	187	-0.26	-0.45 – -0.06	.01
Gibson [43] placebo control and Licciardone [46] no treatment control	6	248	185	-0.35	-0.54 – -0.15	<.001
Median contrasts, Cleary [47] trial excluded	7	310	227	-0.29	-0.47 – -0.12	.001
All contrasts	20	727	520	-0.29	-0.40 – -0.17	<.001

CI denotes confidence interval; OMT, osteopathic manipulative treatment.

*Test for homogeneity, P = .37.

### Stratified analyses

The results of stratified meta-analyses are presented in Table 4. There was a significant reduction in low back pain associated with OMT in trials vs active treatment or placebo control (effect size, -0.26; 95% CI, -0.48 – -0.05; P = .02), independent of fixed-effects or random-effects model specification. There were 243 (3^5 ^× 1^1^) possible contrast combinations based on three contrasts for each of five OMT vs control treatment comparisons [42,43,45,46] and one contrast for another remaining OMT vs control treatment comparison [47]. Both the best-case and worst-case scenarios demonstrated a greater reduction in pain with OMT than active treatment or placebo control, although the worst-case results did not achieve statistical significance. OMT was found to significantly reduce pain in the remaining analyses that limited OMT vs active treatment or placebo control comparisons to one per trial, excluded the Cleary [47] trial, and included all 16 contrasts. The OMT vs no treatment control comparisons were observed in trials in which all subjects received usual low back care in addition to their allocated treatment (ie, OMT and usual care vs only usual care) [44,47]. For these trials, the all-contrasts model (ie, the only model with sufficient contrasts for data synthesis) demonstrated a highly significant reduction in pain with OMT.

**Table 4:** Stratified results.

		**No. of Subjects**				
					
**Model**	**No. of** **Contrasts**	**OMT**	**Control**	**Effect** **Size**	**95% CI**	**P**
**OMT vs. Active Treatment or Placebo Control**						
Median contrasts						
Fixed-effects model*	6	193	142	-0.26	-0.48 – -0.05	.02
Random-effects model	6	193	142	-0.26	-0.48 – -0.05	.02
Best-case scenario	6	174	132	-0.34	-0.57 – -0.11	.004
Worst-case scenario	6	183	134	-0.07	-0.29 – 0.16	.54
Median contrasts, one OMT vs control treatment comparison per trial						
Gibson [43] active treatment	5	154	109	-0.33	-0.58 – -0.08	.01
Gibson [43] placebo control	5	155	115	-0.26	-0.51 – -0.02	.03
Median contrasts, Cleary [47] trial excluded	5	185	138	-0.24	-0.47 – -0.02	.03
All contrasts	16	534	400	-0.21	-0.34 – -0.08	.002
**OMT vs. No Treatment Control**						

All contrasts	4	193	120	-0.53	-0.76 – -0.30	<.001
**Trials Performed in the United Kingdom**						

Median contrasts						
Fixed-effects model*	4	105	84	-0.29	-0.58 – 0.00	.050
Random-effects model	4	105	84	-0.30	-0.63 – 0.02	.06
Best-case scenario	4	105	88	-0.36	-0.64 – -0.07	.01
Worst-case scenario	4	100	83	-0.11	-0.40 – 0.19	.48
Median contrasts, one OMT vs control treatment comparison per trial						
Gibson [43] active treatment	3	66	51	-0.46	-0.83 – -0.09	.02
Gibson [43] placebo control	3	67	57	-0.30	-0.66 – 0.05	.10
Median contrasts, Cleary [47] trial excluded	3	97	80	-0.26	-0.56 – 0.04	.09
All contrasts	10	294	247	-0.23	-0.40 – -0.06	.01
**Trials Performed in the United States**						

Median contrasts						
Fixed-effects model*	4	213	147	-0.31	-0.52 – -0.10	.004
Random-effects model	4	213	147	-0.32	-0.57 – -0.06	.01
Best-case scenario	4	188	132	-0.38	-0.61 – -0.16	.001
Worst-case scenario	4	198	138	-0.22	-0.44 – 0.00	.050
Median contrasts, one OMT vs control treatment comparison per trial						
Licciardone [46] placebo control	3	171	130	-0.24	-0.47 – -0.01	.04
Licciardone [46] no treatment control	3	181	128	-0.36	-0.59 – -0.14	.002
All contrasts	10	433	273	-0.33	-0.48 – -0.18	<.001
**Short-Term Follow-Up**						

Median contrasts						
Fixed-effects model*	5	181	130	-0.28	-0.51 – -0.06	.01
Random-effects model	5	181	130	-0.31	-0.61 – -0.01	.046
Best-case scenario	5	196	142	-0.41	-0.62 – -0.19	<.001
Worst-case scenario	5	181	136	-0.10	-0.32 – 0.12	.38
All contrasts	9	357	258	-0.23	-0.39 – -0.07	.01
**Intermediate-Term Follow-Up**						

Median (all) contrasts						
Fixed-effects model*	7	283	209	-0.33	-0.51 – -0.15	<.001
Random-effects model	7	283	209	-0.36	-0.63 – -0.10	.01
Median contrasts, one OMT vs control treatment comparison per trial						
Gibson [43] active treatment and Licciardone [46] placebo control	5	209	161	-0.31	-0.52 – -0.10	.004
Gibson [43] active treatment and Licciardone [46] no treatment control	5	209	158	-0.45	-0.65 – -0.24	<.001
Gibson [43] placebo control and Licciardone [46] placebo control	5	209	166	-0.25	-0.46 – -0.05	.02
Gibson [43] placebo control and Licciardone [46] no treatment control	5	209	163	-0.39	-0.59 – -0.18	<.001
**Long-Term Follow-Up**						

Median (all) contrasts						
Fixed-effects model*	4	87	53	-0.40	-0.74 – -0.05	.03
Random-effects model	4	87	53	-0.41	-0.82 – -0.01	.046
Median contrasts, one OMT vs control treatment comparison per trial						
Licciardone [46] placebo control	3	55	38	-0.23	-0.65 – 0.19	.28
Licciardone [46] no treatment control	3	55	34	-0.64	-1.08 – -0.20	.01
Median contrasts, Cleary [47] trial excluded	3	79	49	-0.36	-0.72 – 0.01	.054

CI denotes confidence interval; OMT, osteopathic manipulative treatment.

*Tests of homogeneity, P = .45 and P = .06 for active treatment or placebo control, and no treatment control groups, respectively; P = .32 and P = .26 for trials in the United Kingdom and the United States, respectively; and P = .14, P = .06, and P = .28 for short-term, intermediate-term, and long-term follow-up, respectively.

Trials in both the United Kingdom (effect size, -0.29; 95% CI, -0.58 – 0.00; P = .050) and the United States (effect size, -0.31; 95% CI, -0.52 – -0.10; P = .004) demonstrated significant reductions in low back pain associated with OMT. In the sensitivity analyses, effect sizes were generally of comparable magnitude in both countries, although results in American trials consistently achieved statistical significance as a consequence of the larger sample sizes in these trials (Table 4).

**Figure 3:**
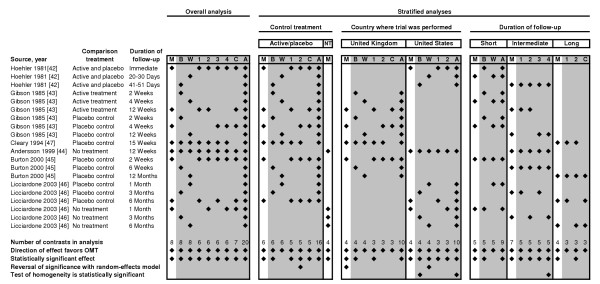
Summary of meta-analysis results. A denotes all-contrasts model; B, best-case scenario model; C, Cleary [47] trial excluded model; M, median contrasts model; NT, no treatment control; OMT, osteopathic manipulative treatment; W, worst-case scenario model. 1, 2, 3, and 4 indicate alternative models restricted to one OMT vs control treatment comparison per trial. A diamond indicates the inclusion of the relevant contrast or observation of the stated result. Sensitivity analyses are shaded in gray. Results are presented for each of the 43 analyses, including the overall meta-analysis, seven stratified meta-analyses, and 35 sensitivity analyses.

There were significant reductions in low back pain associated with OMT during the short-term (effect size, -0.28; 95% CI, -0.51 – -0.06; P = .01), intermediate-term (effect size, -0.33; 95% CI, -0.51 – -0.15; P < .001), and long-term (effect size, -0.40; 95% CI, -0.74 – -0.05; P = .03) follow-up periods. Sensitivity analyses for temporal outcomes demonstrated that intermediate-term results consistently achieved statistical significance, generally because of the greater number of subjects in these analyses (Table 4). The results of the meta-analyses and sensitivity analyses are further summarized in Figure 3.

## Discussion

### Efficacy of osteopathic manipulative treatment

The overall results clearly demonstrate a statistically significant reduction in low back pain with OMT (Figure 2). Further, the meta-analysis results are quite robust as indicated by the comprehensive sensitivity analyses (Figure 3). Stratified meta-analyses to control for moderator variables demonstrated that OMT significantly reduced low back pain vs active treatment or placebo control and vs no treatment control. If it is assumed, as shown in a review [52], that the effect size is -0.27 for placebo control vs no treatment in trials involving continuous measures for pain, then the results of our study are highly congruent (ie, effect size for OMT vs no treatment [-0.53] = effect size for OMT vs active treatment or placebo control [-0.26] + effect size for placebo control vs no treatment [-0.27]).

It has been suggested that the therapeutic benefits of spinal manipulation are largely due to placebo effects [53]. A preponderance of results from our sensitivity analyses supports the efficacy of OMT vs active treatment or placebo control and therefore indicates that low back pain reduction with OMT is attributable to the manipulation techniques, not merely placebo effects. Also, as indicated above, OMT vs no treatment control demonstrated pain reductions twice as great as previously observed in clinical trials of placebo vs no treatment control [52]. Thus, OMT may eliminate or reduce the need for drugs that can have serious adverse effects [44].

Because osteopathic physicians provide OMT to complement conventional treatment for low back pain, they tend to avoid substantial additional costs that would otherwise be incurred by referring patients to chiropractors or other practitioners [54]. With respect to back pain, osteopathic physicians make fewer referrals to other physicians and admit a lower percentage of patients to hospitals than allopathic physicians [1], while also treating back pain episodes with substantially fewer visits than chiropractors [55]. Although osteopathic family physicians are less likely to order radiographs or prescribe nonsteroidal anti-inflammatory drugs, aspirin, muscle relaxants, sedatives, and narcotic analgesics for low back pain than their allopathic counterparts, osteopathic physicians have a substantially higher proportion of patients returning for follow-up back care than allopathic physicians [56]. In the United Kingdom, where general practitioners may refer patients with spinal pain to osteopaths for manipulation, it has been shown that OMT improved physical and psychological outcomes at little extra cost [57].

In our study, the effect sizes for OMT in the United Kingdom, where osteopaths are not licensed physicians, were generally comparable to those in the United States, where OMT is provided by licensed physicians. This consistency suggests that the results truly reflect the effects of OMT itself, and not other elements of low back care. It is not surprising that osteopaths in the United Kingdom achieved pain reduction with OMT similar to that of their physician counterparts in the United States. The training of osteopaths in the United Kingdom is highly focused on OMT, whereas osteopathic physicians undertake a medical curriculum that necessarily relegates OMT to one of many therapeutic approaches, albeit a fundamental one for osteopathic practitioners. Regardless of the career training path of the provider, it appears that OMT achieves clinically important reductions in low back pain.

### Potential limitations

There are several potential limitations of this study that should be addressed. First, as with any meta-analysis, the individual trials varied somewhat with respect to methodology, including trial setting, subject characteristics, OMT and control treatment interventions, and pain measures (Table 1). Such heterogeneity has been commonly observed in previous meta-analyses of spinal manipulation, including a recent meta-analysis performed in collaboration with the Cochrane Back Review Group [31]. The latter study addressed potential heterogeneity by presenting stratified results according to chronicity of low back pain, type of control group, and duration of follow-up. This approach is analogous to the methods used in our study. Further, it should be noted that the assumption of homogeneity among trials was not rejected statistically in any of our eight overall or stratified median contrasts meta-analyses.

Second, because five trials each included repeated pain measures and two trials each included two control treatments, there was no unique set of independent outcomes for meta-analysis. Such interdependencies were noted to be a problem in an early meta-analysis of spinal manipulation [15]. We used the median contrasts method to address this problem because the median outcome represents an observed outcome that is easy to compute and is less vulnerable to extreme observations than other measures of central tendency. Further, sensitivity analysis was used to assess the range of possible combinations of outcomes. Thus, for the overall meta-analysis, there were 729 potential contrast combinations. Of these, both the best-case and worst-case scenarios demonstrated statistically significant results favoring OMT, thereby providing unequivocal evidence for the efficacy of OMT. Robust findings were also observed for trials performed in the United States and for intermediate-term outcomes.

Third, because there were a relatively small number of eligible trials, there were not sufficient contrasts for certain analyses and some results were imprecise. The latter phenomenon likely obviated the statistical significance of some results. Nevertheless, it is important to note that the direction of results favored OMT in each of the 43 meta-analyses and sensitivity analyses presented herein (Figure 3).

Fourth, there exists the possibility that the results of unpublished trials of OMT for low back pain may have altered significantly the conclusions of this study. To address this issue, we performed file drawer analysis by computing the fail-safe N [58]. This represents the number of unpublished trials of OMT for low back pain that would have met our inclusion criteria, and that also would have demonstrated an effect size averaging ≥ -0.10, which is assumed to reflect clinically insignificant levels of pain reduction. A total of 16 unpublished trials (assuming one control group per trial) with, in the aggregate, clinically insignificant pain reduction outcomes would have been needed to obviate the significance of our results. Only recently has government funding for research in the area of complementary and alternative medicine become more widely available, in response to the public's interest in such treatments. Historically, it is highly unlikely that 16 trials of OMT for low back pain would have been sponsored, conducted, and subsequently not published.

Finally, this study focused only on the efficacy of OMT with respect to pain outcomes. Generic health status, back-specific function, work disability, and back-specific patient satisfaction are other recommended outcome domains [59] that were not assessed because the included OMT trials did not consistently report these data.

## Conclusion

The present study indicates that OMT is a distinctive modality that significantly reduces low back pain. The level of pain reduction is greater than expected from placebo effects alone and persists for at least three months. Additional research is warranted to elucidate mechanistically how OMT exerts its effects, to determine if OMT benefits are long lasting, and to assess the cost-effectiveness of OMT as a complementary treatment for low back pain.

## Competing interests

The author(s) declare that they have no competing interests.

## Authors' contributions

JCL, AKB, and LNK conceived and designed the study. LNK performed the literature searches. JCL and AKB extracted the data. JCL performed the statistical analyses. JCL, AKB, and LNK interpreted the data and drafted the manuscript. JCL will act as guarantor for the paper. The guarantor accepts full responsibility for the conduct of the study, had access to the data, and controlled the decision to publish. All authors approved the final manuscript.

## Pre-publication history

The pre-publication history for this paper can be accessed here:



## Supplementary Material

Additional File 1 this file provides the timetable, databases, and search terms used to identify relevant studies for the meta-analysis. Click here for file
