# Supplementary material for: Osteopathic manipulative treatment for low back pain: a systematic review and meta-analysis of randomized controlled trials
Source: BMC Musculoskelet Disord. 2005 Aug 4;6:43. doi: 10.1186/1471-2474-6-43 (PMC1208896; doi:10.1186/1471-2474-6-43)
Supplement: Additional File 1 — this file provides the timetable, databases, and search terms used to identify relevant studies for the meta-analysis. [file 1471-2474-6-43-S1.doc]

**Osteopathic manipulative treatment for low back pain:**

**a systematic review and meta-analysis**

**of randomized controlled trials**

**Appendix**

**Search strategy**

A review of the literature was performed in November 2002 to produce a bibliography of articles, abstracts, and research reports in which osteopathic manipulative treatment (OMT) of any condition or disease was studied in a randomized controlled trial. The following databases were searched: MEDLINE (OVID), OLDMEDLINE, MANTIS, OSTMED, Alt Health Watch, ClinicalTrials.gov, CRISP, and the Cochrane Central Register of Controlled Trials. Additionally, searches were performed in EMBASE and SciSearch in December 2002.

We attempted to acquire a comprehensive retrieval (high recall) of the literature, despite the potential retrieval of non-relevant articles. An experienced medical librarian and database searcher developed the strategies and performed all the searches described herein. The librarian then reviewed the initial retrieval and eliminated obvious false drops and duplicates.

A brief description of each database at the time of the original search is provided below. Each description is followed by the applicable search strategy because each database has its own unique criteria for searching, including controlled vocabulary (thesaurus), words and phrases in the title and abstract, languages, publication types, years covered, population studied, and ability to truncate terms and combine concepts using Boolean logic. Each database was searched using a unique interface. All searches were limited to the English language.

MEDLINE is the premier biomedical bibliographic database, produced by the National Library of Medicine (NLM). NLM indexes over 4600 worldwide life science journals. MEDLINE includes over 12 million references from 1966 to the present. Indexers assign Medical Subject Headings (MeSHs), NLM’s controlled thesaurus of descriptors. The database can be searched using MeSH terms, author name, title word, text word, journal name, phrase, or any combination of these. Most of the references starting in 1975 also contain an abstract. MEDLINE was searched using the OVID search interface. “Manipulation, osteopathic” became a MeSH term in 2002. To include earlier references, the following search strategy was used:

1. manipulation, osteopathic (MeSH) or OMT (text word) or osteopath$ (text word)
2. osteopathic medicine (MeSH) and manipulation, orthopedic (MeSH)
3. 1 or 2
4. 3 and randomized controlled trial (publication type)

OLDMEDLINE covers the same biomedical literature as MEDLINE for the years 1957 to 1965. Access to this database is by key words, original MeSH terms (not updated), and text phrases. OLDMEDLINE was searched with the NLM Gateway, using “osteopath* or OMT” as search terms. "Randomized controlled trial" as a publication type was not available during this time period.

The Excerpta Medica Database (EMBASE) is a biomedical and pharmacological resource providing access to the most up-to-date information about medical and drug-related subjects. It covers the literature from 1974 to the present. EMBASE provides access to periodical articles from more than 3,700 primary journals from approximately 70 countries. Additionally, 350 journals are screened for drug articles. The following search strategy was used:

1. Osteopath? Or OMT
2. random? And trial? ?
3. 1 and 2

Manual, Alternative and Natural Therapy (MANTIS) is a bibliographic database that includes health care disciplines not significantly represented in the major biomedical databases. The database contains references from more than 1,000 journals, with preference given to peer-reviewed journals. Approximately 70% of the references have abstracts. Searchable subject headings include MeSH terms, as well as a specialized supplemental controlled vocabulary in the areas of alternative medicine. The search strategy for this database combined “randomized” with “osteopath* or OMT.”

OSTMED is a bibliographic database providing access to the osteopathic medicine literature. It is currently in development by the Gibson D. Lewis Health Science Library of the University of North Texas Health Science Center at Fort Worth in conjunction with the Kirksville College of Osteopathic Medicine's A.T. Still Memorial Library. OSTMED is the product of a 5-year project sponsored by the American Osteopathic Association (AOA) and the American Association of Colleges of Osteopathic Medicine (AACOM). The OSTMED database currently contains some 30,000 citations and abstracts of journal articles and meeting abstracts. It covers the literature since 1892. Subject access is through keywords, MeSH terms, and a structured thesaurus of unique osteopathic terms derived from the "AACOM Glossary of Osteopathic Terminology" and other resources. The database was searched using “randomized controlled trials” as a MeSH term or as a text phrase, and “randomized clinical trials” as a text phrase, and the resulting list was reviewed to identify those references actually using OMT in the study.

Alt Health Watch focuses on complementary, holistic, and integrated approaches to health care and wellness. Alt Health Watch includes indexing and full-text articles from over 170 journals, magazines, newsletters, reports, and conference proceedings, as well as hundreds of pamphlets, special reports, and book excerpts. It was searched through the Ebsco interface, using the term “randomized” combined with “osteopath* or OMT.”

SciSearch is an international, multidisciplinary index and cited reference science database to the literature of science, technology, biomedicine, and related disciplines. SciSearch indexes all significant items (articles, review papers, meeting abstracts, letters, editorials, book reviews, correction notices, etc.) from approximately 4,500 major scientific and technical journals. Some 3,800 of these journals are further indexed by the references cited within each article, allowing for citation searching. SciSearch covers the period from 1974 to the present. The search strategy for this database combined the term “randomized” with “osteopath* or OMT.”

ClinicalTrials.gov is a database of clinical research protocols funded by the NIH, other federal agencies, and the pharmaceuticals industry. Computer Retrieval of Information on Scientific Projects (CRISP) is a biomedical database system with information on research projects supported by the Department of Health and Human Services. Both databases were searched using the terms “osteopath* or OMT.”

The Cochrane Central Register of Controlled Trials (CCTR) is a component of the Cochrane Evidence-Based Medicine Reviews Collection. It is a bibliographic database of definitive controlled trials identified by the contributors to the Cochrane Collaboration. CCTR contains over 300,000 bibliographic references to controlled trials in health care. Cochrane groups and other organizations contribute their specialized registers, and together with references to clinical trials identified in MEDLINE and EMBASE, form the CCTR database. CCTR was searched using the terms “osteopath$” or “OMT.”

In August 2003, searches of the databases that had previously led to the identification of substantial numbers of citations were updated. These included MEDLINE, OSTMED, EMBASE, MANTIS, and the Cochrane Central Register of Controlled Trials. The same search strategies described above were used to cover the most recent eight to nine month time period.

The subsequent criteria for selection of trials to be included in the meta-analysis are described in the Methods section, and the flowchart of trials is presented in Figure 1.
